# Applying a modified streamlined disease risk analysis framework to a platypus conservation translocation, with special consideration for the conservation of ecto- and endoparasites

**DOI:** 10.1016/j.ijppaw.2024.100948

**Published:** 2024-05-23

**Authors:** Jessica Whinfield, Kristin Warren, Larry Vogelnest, Rebecca Vaughan-Higgins

**Affiliations:** a The Harry Butler Institute, Murdoch University, Murdoch, Western Australia, Australia; b Taronga Conservation Society Australia, Mosman, New South Wales, Australia

**Keywords:** Platypus, Wildlife health, Conservation translocation, Parasite conservation, Disease risk analysis

## Abstract

Platypuses are the world's most evolutionarily distinct mammal and have several host-specific ecto- and endoparasites. With platypus populations declining, consideration should also be given to preserving these high conservation priority parasites alongside their charismatic host. A disease risk analysis (DRA) was performed for a platypus conservation translocation, using a modified streamlined methodology that incorporated a parasite conservation framework. DRA frameworks rarely consider parasite conservation. Rather, parasites are typically considered myopically in terms of the potential harm they may cause their host. To address this, a previously proposed parasite conservation framework was incorporated into an existing streamlined DRA methodology. Incorporation of the two frameworks was achieved readily, although there is opportunity for further refinement of this process. This DRA is significant as it is the first performed for any monotreme species, and implements the emerging approach of balancing the health and disease risk of the host with parasite conservation.

## Highlights

• First disease risk analysis (DRA) performed for a monotreme species.
• Incorporates a parasite conservation framework and a streamlined DRA methodology.
• Real-world example considering both host health and parasite conservation.
• Consideration of host-specific platypus parasites as high conservation value.

## Graphical abstract

**Figure:**
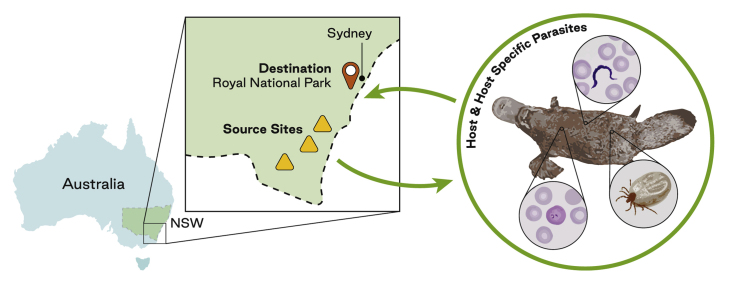

## 1 Introduction

There is growing recognition of the importance of health management in conservation translocations, and the potential for health to influence their outcomes (Beckmann et al., 2022). In response, health management is increasingly being embedded within the planning, execution, and monitoring of conservation translocations. The disease risk analysis (DRA) process is a structured and evidence-based decision-making methodology that is commonly used for this purpose (Jakob-Hoff et al., 2014). Performing a DRA prior to conservation translocations is considered best-practice, and, in some jurisdictions, is required prior to receiving Government approval for translocations (Department of Planning, Industry and Environment NSW, 2019). The most frequently applied DRA methodology is that described by the International Union for Conservation of Nature (IUCN) (Jakob-Hoff et al., 2014). However, despite its widespread use, and being considered the gold-standard methodology (Vaughan-Higgins et al., 2021), there are limitations to the DRA methodology described by the IUCN.

One limitation of the IUCN DRA framework (Jakob-Hoff et al., 2014) is that it does not embed parasite conservation (Carlson et al., 2020). Ecto- and endoparasites are ubiquitous, ecologically important, and often lacking in conservation protection (Carlson et al., 2020). Beyond the intrinsic value of parasites, there are potential host benefits to ecto- and endoparasite conservation. For example, parasites act as critical food web links, and small effects on the diversity of parasites and parasite community structures can have larger and unpredictable impacts: these range from effects on host immunity and health, to consequences for the transmission of diseases, and even ecosystem-level impacts (Esser et al., 2019; Carlson et al., 2020). In some scenarios, translocation success may be reduced through host health being impacted by the disruption of host-parasite relationships and the perturbance of parasite infracommunities (Northover et al., 2018). Whilst parasite conservation is not a new concept (Gompper and Williams, 1998; Pérez et al., 2006; Pizzi, 2009), it has rarely been purposefully integrated into the methodology of DRAs. DRA frameworks typically have a narrow focus on host health, rather than balancing this important objective alongside parasite conservation and ecosystem richness. This is highlighted by the language of the IUCN's DRA framework (Jakob-Hoff et al., 2014), where all infectious organisms are termed ‘hazards’, irrespective of the level of threat they pose to the host. The incorporation of parasite conservation requires the focus of the DRA to change from minimising the health risks of vertebrates, to the broader objective of preserving symbiont assemblages and microorganisms, thereby promoting ecosystem diversity and health.

There are multiple challenges to the conservation of ecto- and endoparasites. One challenge is that often the conservation status of parasites is not formally defined, and therefore assessments need to be made based on an understanding of parasite ecology (Lymbery and Smit, 2023). For example, host-specific parasites may be more endangered than their hosts, as a small host population size may reduce transmission levels below the threshold required for maintenance of the parasite population (Lymbery and Smit, 2023). Another challenge is that, by design, some conservation practices developed to protect hosts may directly harm their parasite communities, such as disinfection protocols (Carlson et al., 2020). Additionally, parasites can negatively impact host health, which can in turn reduce the chance of the success of conservation actions (Beckmann et al., 2022). Translocations may disrupt host-parasite community relationships in complex and unexpected ways (Rideout et al., 2017; Northover et al., 2018). Therefore, it is important that conservation strategies, including translocations, consciously consider whether it is possible to achieve host-health objectives without unduly impacting parasite conservation, remembering that the host itself is habitat (Pérez et al., 2006).

Another limitation of the IUCN's DRA methodology (Jakob-Hoff et al., 2014) is the significant input of time and labour required for its application (Vaughan-Higgins et al., 2021). To make the DRA process less intensive for practitioners, several modified approaches to performing a DRA have been described (Sainsbury et al., 2012; Vaughan-Higgins et al., 2021). These modified approaches have a variety of benefits, including streamlining the DRA process and accordingly significantly reducing the time taken to complete DRAs.

The streamlined methodology described by Vaughan-Higgins et al. (2021) was used to perform a DRA for a platypus (*Ornithorhynchus anatinus*) conservation translocation. Platypuses are native to eastern Australian freshwater ecosystems (Bino et al., 2019) and listed by the IUCN as near threatened with a decreasing population trend (Woinarski and Burbidge, 2016). This decline is predicted to continue as threatening processes accelerate, including: changing land use, specifically urbanisation and agriculture (Brunt et al., 2021); the intensive management of natural resources, including the construction of dams (Hawke et al., 2021); and extreme climatic events exacerbated by anthropogenic climate change, including droughts, floods, and fires (Griffiths et al., 2020). The resilience of platypus populations to these processes could be diminished by their limited ability for overland dispersal (Furlan et al., 2013). This may result in platypuses failing to re-establish in areas following local extinctions, especially when there is poor or absent waterway connectivity to other platypus populations (Hawke et al., 2021). For this reason, conservation translocations, particularly assisted reintroductions, may become an increasingly important strategy for platypus conservation.

Platypuses are recognised as the world's most evolutionarily distinct mammal species (Isaac et al., 2007) and there are several host-specific ecto- and endoparasites that have evolved alongside them. These host-specific parasites include the tick *Ixodes ornithorhynchus* (Roberts, 1970; Kwak et al., 2018), and the haemoprotozoans *Theileria ornithorhynchi* (Mackerras, 1959; Paparini et al., 2015) and *Trypanosoma binneyi* (Paparini et al., 2014; Thompson et al., 2014). There has previously been minimal consideration for conserving these ecto- and endoparasites alongside platypuses. Our understanding of platypus health, including the ecology and epidemiology of these host-specific infectious organisms (Šlapeta et al., 2017), is hampered by the cryptic nature of platypuses as semi-fossorial, semi-nocturnal, and semi-aquatic animals. Whilst gaps that exist in our current understanding must be acknowledged in the DRA process, they should prohibit neither the performance of a DRA, nor the consideration of parasite conservation.

The DRA described in this paper was performed for the conservation translocation of wild platypuses to Royal National Park (NP), on Dharawal country, approximately 30 km south of Sydney, New South Wales (NSW), Australia. Platypuses have historically occurred in Royal NP however local extirpation was confirmed through extensive eDNA testing of freshwater systems within the NP prior to the translocation. The assisted reintroduction involved the translocation of 10 wild platypuses from several sites in southeast NSW. This paper outlines the DRA process which was undertaken prior to the translocation of the platypuses, in accordance with Government requirements (Department of Planning, Industry and Environment NSW, 2019). To the authors knowledge, this is the first DRA performed for a monotreme species.

There are two primary aims of this article. Firstly, to describe the application, benefits, and limitations of embedding a previously proposed parasite conservation template (Carlson et al., 2020) within an existing DRA framework (Jakob-Hoff et al., 2014; Vaughan-Higgins et al., 2021). Secondly, to describe the application of the streamlined DRA process described by Vaughan-Higgins et al. (2021) to a platypus translocation with a unique set of challenges. Indeed, all four factors nominated in Sainsbury and Vaughan-Higgins (2012) as increasing the complexity of completing a DRA were present for the platypus DRA presented here. As more translocations, and DRAs, are performed, it is critical that there is fine-tuning of the DRA process. Towards this goal, it is important to share the learnings from the challenges faced in performing DRAs under different circumstances. By doing so, we can better achieve the overarching goal of conserving and restoring ecosystems whilst mitigating the ubiquitous health risks that accompany translocations.

## 2 Materials and methods

A DRA was performed based on the streamlined approach described by Vaughan-Higgins et al. (2021), except where explicitly stated to have deviated from this method. The below subheadings reflect (with minor adaptation) the IUCN's five-step DRA process (Jakob-Hoff et al., 2014), from which the Vaughan-Higgins et al. (2021) streamlined approach was adapted.

Step 1: Problem description

The first step in the IUCN's DRA process is the problem description: “the process of describing and justifying the problem or questions” (World Organisation of Animal Health & International Union for the Conservation of Nature, 2014). A problem description was developed, which: 1) provided background and context; 2) identified the goal, scope, and focus of the DRA; 3) stated assumptions and limitations; and 4) established a statement on the acceptable level of risk, developed with input from key stakeholders. Three biotic groupings, termed hazard groups, were defined for consideration of their disease risk (Table 1). Likelihood and consequence were also defined (Table 1), and, in a departure from the methodology of Vaughan-Higgins et al. (2021), these terms were defined separately for the three hazard groupings.

Step 2: Hazard identification and prioritisation

**Table 1:**
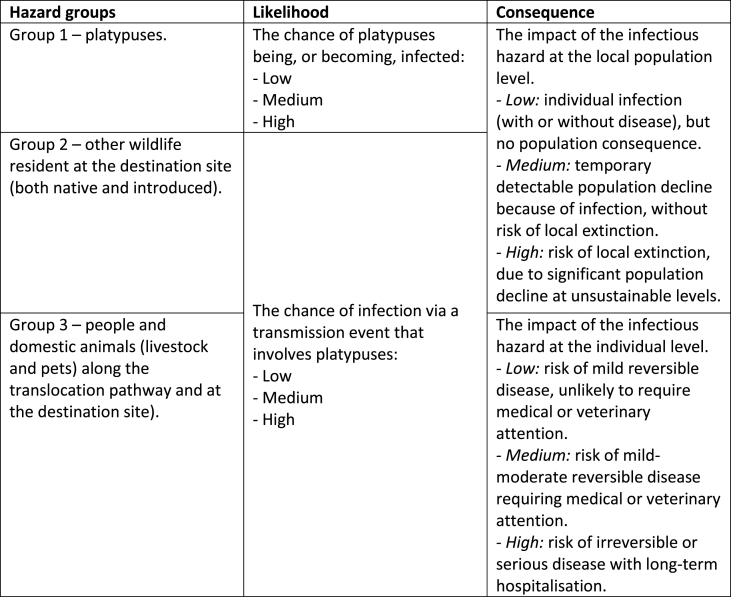
Definitions of likelihood and consequence for the three hazard groupings.

Hazard identification and prioritisation was performed. This was achieved through a desktop review of the primary and secondary literature. For each potential infectious hazard identified through this review, a hazard summary was produced. The hazard summary included information on the following: potential host species; geographic range; possible transmission pathways; and possible impacts and consequences. This information was then used to qualitatively determine the likelihood and consequence ratings for each of the three hazard groupings. Importantly, the likelihood of people becoming infected was assessed based on the stated assumption that all persons in direct contact with platypuses and their immediate environment would undertake standard hygiene practices, such as handwashing and covering skin abrasions with waterproof dressings. For each hazard group, the likelihood and consequence ratings were then combined to generate an overall risk rating using a risk matrix (Table 2). This facilitated hazard prioritisation: only hazards with an overall risk rating of medium or high for any hazard population were subjected to a comprehensive risk assessment.

Step 3: Comprehensive risk assessment, incorporating ecto- and endoparasite conservation

**Table 2:**
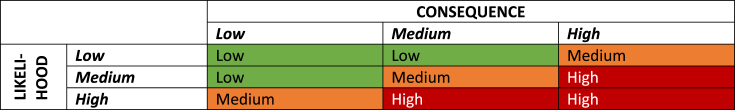
Risk matrix for generating overall risk ratings.

**Fig. 1:**
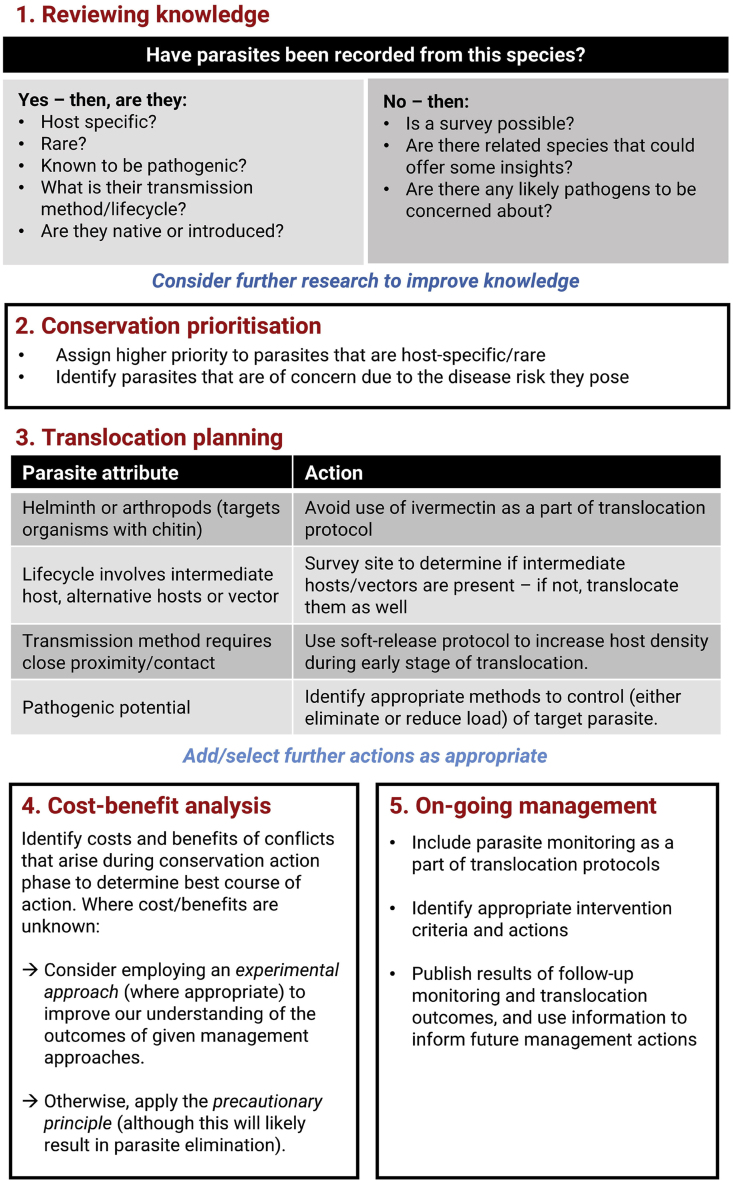
An outline of how parasite conservation can be considered in translocation planning, reproduced from Carlson et al. (2020).

The comprehensive risk assessment expanded on the information collated during the hazard identification and prioritisation process. When the subject of the comprehensive risk assessment was an ecto- or endoparasite, we applied the five-step process proposed by Carlson et al. (2020) for considering metazoan parasite conservation in translocation planning (Fig. 1). The inclusion of the parasite conservation process was a departure from the methodology described in Vaughan-Higgins et al. (2021). The process developed by Carlson et al. (2020) was designed to be applied simultaneously with the standard DRA process, and indeed, no significant adaptations were required to combine and apply the two processes. Whilst the Carlson et al. (2020) framework was developed for metazoan parasites, we applied it to both protozoal and metazoan species. Through this process, simplified parasite conservation prioritisation occurred, with platypus-dependent/host-specific parasites assigned a higher conservation value, as per Carlson et al. (2020; Fig. 1, step 3).

Step 4: Risk management

For each full risk assessment performed, risk management options were produced. These aimed to both reduce the risk for the three hazard groupings (where indicated), and minimise threats to the survival of ecto- and endoparasites assessed as being of high conservation value. These risk management options were collated to form a list of recommended risk mitigation strategies.

Step 5: Implementation and review

The DRA was provided in-full to key stakeholders in the Royal NP platypus translocation. These stakeholders performed decision making around the implementation of the recommended risk mitigation strategies. An abridged version of the DRA, edited for brevity and clarity, was submitted as part of the translocation proposal, in accordance with NSW legislation (Department of Planning, Industry and Environment NSW, 2019). Additionally, the DRA was shared on request with other groups involved in the management of wild platypuses.

Through the DRA process, critical knowledge gaps were identified. In response, we developed research questions, and sampling and biobanking protocols. The DRA is a cyclical, iterative process (Jakob-Hoff et al., 2014), and thus the results of these ongoing investigations will be used to revise the DRA.

## 3 Results

The problem description that was produced is shown in Table 3.

**Table 3:** Problem description for the assisted reintroduction of platypuses to Royal NP.

Background and context	- Platypuses listed by IUCN as near threatened (Woinarski and Burbidge, 2016); vulnerable to local extinctions due to limited overland dispersal capacity (Hawke et al., 2021). - Threatening processes include habitat loss, river regulation, and extreme climatic events (Bino et al., 2019). - DRA to be performed for an assisted reintroduction of platypuses to Royal NP, with the translocation of 10 wild founders from multiple source sites in southeast NSW. - Platypuses locally extinct within Royal NP (extensive eDNA testing conducted), but historically present. - Platypus translocations have been sporadically performed (Furlan et al., 2012), however, no DRA previously produced for any monotreme species.
DRA goal	- To develop an infectious disease risk management strategy for platypuses translocated from multiple wild source populations to a single destination (Royal NP). - Achieved through a systematic, structured, and evidence-based analysis of relevant available information.
DRA scope	- Reviewed materials restricted to published information on both wild and captive platypuses. - Risk of infectious diseases affecting translocated animals at destination environment. - Risk of translocated platypuses acting as vectors for infectious diseases to people, domestic animals, and existing vertebrate fauna, both along the translocation pathway and at the destination environment.
DRA focus	- Known infectious pathogens of platypuses, and select infectious organisms with broad host ranges that platypuses may theoretically be susceptible to. - Non-infectious hazards are not the focus of the DRA.
Assumptions	- Platypuses susceptible to both those infectious health hazards previously reported in the species, and some other infectious organisms with broad host ranges not previously reported in platypuses. - No other novel, unknown, or as-yet undetermined infectious disease risks for platypuses. - Robust decision making related to infectious disease can occur by using the available data, expert opinions, and analytical and decision-making processes. - Platypuses locally extinct in Royal NP prior to the translocation. - Hazard prioritisation and full risk assessments performed with the assumption that standard hygiene practices followed by all people in direct contact with platypuses, their excreta, and their environment. Standard hygiene practices include thorough and regular hand washing, and covering skin abrasions with waterproof dressings.
Limitations	- Limited understanding of disease ecology in platypuses, especially mainland populations. - Fundamental information lacking for many infectious hazards of platypuses, including: pathogen prevalence, pathogen distribution, pathogen variation between populations, and infection impact. - Pathogen screening tests may not be validated in platypuses. - Evolutionary uniqueness of the platypus (Isaac et al., 2007) means extrapolation of health and disease information from better-studied species should be undertaken with caution. - Due to time and accessibility constraints, the DRA was a desktop process compiled by a single author and could not incorporate unpublished information such as medical records. - The location of source populations was not confirmed when the DRA was performed.
Acceptable level of overall risk for each hazard population	Group 1, platypuses: low to medium. Group 2, wildlife at the destination site: low to medium. Group 3, people and domestic animals: low.

Through the hazard identification and prioritisation process, 23 hazards were identified (Table 4). An example of the hazard summaries that were generated for each of the 23 identified hazards is shown in Table 5, using the example of *Ixodes ornithorhynchi*. 17 infectious hazards were considered low risk to all hazard groups; six infectious hazards were considered medium risk to one or more hazard population; and no infectious hazards were considered high risk to any population (Table 4). A full risk assessment was performed on all six of the infectious hazards that were considered a medium risk. These hazards were *Leptospira* spp., *Mucor amphibiorum*, *Theileria ornithorhynchi*, *Trypanosoma binneyi, Toxoplasma gondii,* and *Ixodes ornithorhynchi.* An example of a full risk assessment is shown in Table 6**,** again using the example of *Ixodes ornithorhynchi.* Finally, based on these results, risk mitigation recommendations were collated (Table 7). Some, but not all, recommendations were adopted based on the outcomes of discussions with stakeholders.

**Table 4:**
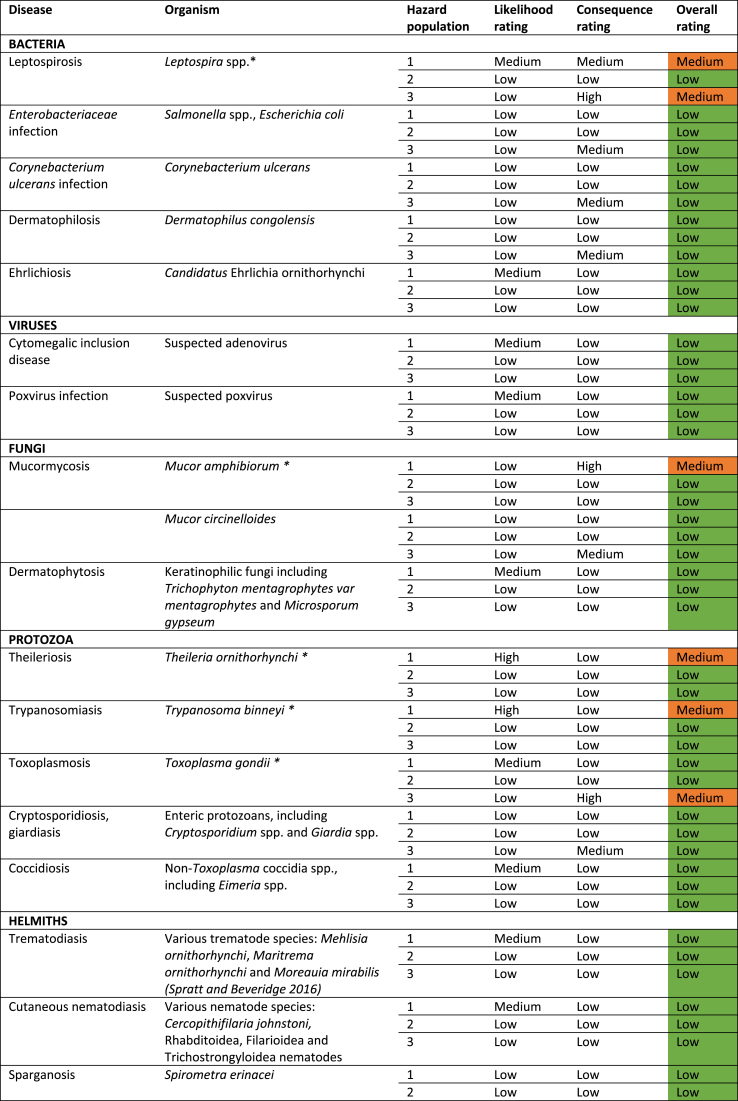

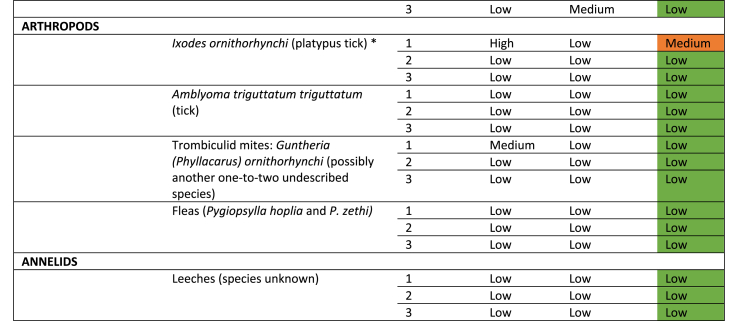
Summarised results of the infectious hazard identification and prioritisation process. * - infectious hazard subjected to a full risk assessment.

**Table 5:**
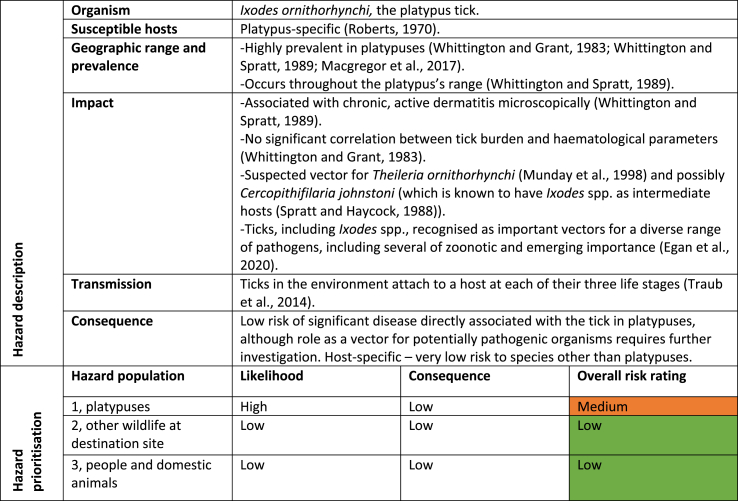
Hazard summary and prioritisation example, *Ixodes ornithorhynchi* (Egan et al., 2020; Traub et al., 2014).

**Table 6:** Example of a comprehensive risk assessment incorporating the parasite conservation framework (Carlson et al., 2020), performed for *Ixodes ornithorhynchi.* Superscript numbers correspond to the steps described in Fig. 1.

Organism^1.^	*Ixodes ornithorhynchi,* the platypus tick.
Host species^1.^	Platypus-specific (Roberts, 1970).
Range^1.^	Endemic (Roberts, 1970). Highly prevalent in platypuses across their range (Whittington and Grant, 1983; Whittington and Spratt, 1989; Macgregor et al., 2017).
Pathogenicity^1.^	- Field studies conducted on apparently healthy platypuses found no association between *I. ornithorhynchi* burdens and haematological parameters, including white and red blood cell counts (Whittington and Grant, 1983). - Has been associated with chronic, active dermatitis microscopically (Whittington and Spratt, 1989). - Ticks well-recognised as important vectors for blood-borne pathogens (Krige et al., 2019). *I. ornithorhynchi* suspected as vector for *Theileria ornithorhynchi* (Munday et al., 1998). Possible vector for *Cercopithifilaria johnstoni* (which is known to have *Ixodes* spp. as intermediate hosts (Spratt and Haycock, 1988)). May be a vector for yet-to-be-described infectious organisms. - Anecdotally, above average tick burdens (hundreds-thousands) have been reported on juveniles in poor body condition and with poor waterproofing. However, whether the ticks are the primary cause of pathology, or reflect a compromised host not effectively grooming, is unclear.
Transmission method^1.^	*Ixodid* spp. ticks have a three-host life cycle. After seeking and feeding from a host, drop off into the environment to develop before finding next host (Tahir et al., 2020).
Risk evaluation^2.^	Risk of negative consequences from *I. ornithorhynchi* is low. Conservation priority is high, based on framework proposed by Carlson et al. (2020).
Conservation prioritisation^2.^	Unlisted by the IUCN (IUCN, 2023). As a host-specific parasite, conservation status likely the same as host (platypuses listed by IUCN as near threatened (Woinarski and Burbidge, 2016)), or higher (Lymbery and Smit, 2023). Considered high conservation priority.
Risk management options^3-5.^	- No direct parasite management recommended. If ticks must be removed during the translocation for research purposes, recommend subsampling the population of ticks on each host, rather than removing all ticks. - Post-translocation platypus surveys should assess presence/absence and relative abundance of ticks. - Recommended that only platypuses considered in reasonable body condition are translocated, reducing risk of subclinical infections becoming clinical. Decisions around appropriate body condition to be made in consultation with stakeholders. - Only translocate sub-adult or adult animals, as juveniles (anecdotally) may have higher incidence of disease associated with some infections, including *I. ornithorhynchi.* - Other management options that could be considered, but are not recommended, include: 1) treat platypuses with parasiticides to remove ticks; 2) manually remove ticks during pre-translocation health checks.

**Table 7:** Collated risk mitigation recommendations provided to key stakeholders.

Select source populations with minimal geographical barriers, both between other source populations, and the destination site.
Maintain strict biosecurity measures during the translocation. This includes removing dirt and other gross contaminates from equipment, and disinfecting with fungicides such as dimethyl dodecyl ammonium chloride (to reduce the risk of spread of environmental fungi that may be present, such as *Mucor* species).
Perform post-translocation monitoring and surveillance, including of ecto- and endoparasites. This will increase the chance of detecting diseases that may arise in the population post-translocation, and enables the monitoring of the impact of translocation on the complete biological package.
Actively control feral species (including dogs, foxes, cats, and deer) within Royal NP. This will reduce the risk associated with both infectious hazards (deer – *Leptospirosis* spp.; cats – *Toxoplasma gondii*) and non-infectious hazards (dogs and foxes – predation; deer – bank erosion).
Do not directly manage subclinical parasite infections.
Minimise stress during the translocation. This should include minimising exposure to stressful stimuli such as loud noises, maintaining animals at optimal temperatures, and minimising handling.
All animals proposed for translocation should undergo a thorough physical examination under anaesthesia by an experienced wildlife veterinarian prior to translocation, including an assessment of haematological parameters. Animals with abnormalities detected may be excluded from the translocation at the discretion of the veterinarian. Animals with clinical abnormalities identified should be treated for the benefit of individual health and welfare. Sampling for research purposes may also be beneficial, if appropriate.
Platypuses in poor body condition should be excluded from the translocation.

## 4 Discussion

This platypus DRA adds to the growing number of DRAs performed for Australian wildlife (Jakob-Hoff et al., 2015, 2016; Cowen et al., 2023). The streamlined DRA approach described by Vaughan-Higgins et al. (2021) (with minor modifications) proved to be a robust and flexible tool for performing the platypus DRA. In particular, the hazard prioritisation process – which results in hazards with overall low risk ratings being excluded from a full disease risk assessment – was beneficial in terms of reducing workload whilst still effectively managing risk. We also found that the parasite conservation framework developed by Carlson et al. (2020) integrated well into the existing DRA structure, requiring minimal additional effort to implement. Crucially, it enabled the DRA scope to expand beyond a tool focused exclusively on the health of the host, instead transforming the DRA into a tool capable of considering parasite conservation alongside host health objectives.

An unanticipated benefit of the DRA process was its usefulness in addressing the same knowledge gaps that presented a limitation to its completion. By identifying fundamental gaps in the current understanding of platypus disease ecology and epidemiology, the DRA enabled research questions to be developed. The capture of animals as part of the translocation presented a valuable opportunity for sample collection to address these research questions. To this effect, two examples of investigations that have evolved from the platypus DRA are a study into the epidemiology of Leptospirosis in the species, and a survey of the protozoal parasites of NSW platypuses. We recommend practitioners consider and maximise, where possible, the opportunities for addressing knowledge gaps afforded by both the DRA and the larger translocation process.

Communication is always central to the DRA process (Jakob-Hoff et al., 2014), and discussions with stakeholders were critical for assessing the feasibility of implementing the proposed risk mitigation strategies. The most significant of these discussions was around balancing the risk and benefit of using multiple source populations, which were separated by geographical barriers both from other source populations and the destination. Translocations that occur across geographical and ecological barriers are recognised as carrying an increased disease risk (Bobadilla Suarez et al., 2017). A species-specific assessment for the presence of these barriers must be made, as what constitutes a barrier may vary widely between species. For platypuses, there is evidence that geographical barriers may readily impact movement, even when populations are in close proximity: a genomics study found no evidence of platypus migration or recent admixture between two physically close locations, a finding attributed to steep terrain separating the rivers (Martin et al., 2018). No thorough screening for infectious hazards could be performed prior to the translocation, and therefore the degree of variation in pathogen profiles between the proposed source populations was unknown. The perceived increased disease risk resulting from the presence of geographical barriers had to be balanced with the benefits of having multiple source populations. These benefits included increasing the genetic diversity of founders, and reducing the impact on the source populations. Ultimately, based on stakeholder discussions, it was decided that the theoretically increased disease risk did not outweigh the benefits of using multiple, but potentially geographically isolated, populations. Further supporting this decision was the assessment that the overall disease risk of the translocation was relatively low. This was, in part, because the translocated animals would likely form a closed population due to limited waterway connectivity to other platypus populations. Thus, any disease outbreaks in the translocated animals were considered unlikely to spread to other platypus populations.

Formally considering ecto- and endoparasite conservation within the DRA process remains relatively uncommon, and whilst it was included in this platypus DRA, there were limitations to our approach. Firstly, we only applied the parasite conservation framework to the four parasites for which a full disease risk assessment was performed*.* We propose that at the hazard identification and prioritisation stage (section 2, step 2), an assessment of parasite conservation priority should be made, with all high conservation priority organisms subjected to a full risk assessment. Secondly, the approach we took remained host-centric, with a passive approach to parasite conservation. For example, a risk mitigation action was to not manage subclinical parasite burdens. Going forward, we recommend a more active approach, where translocating high conservation value parasites alongside their host is a project objective, a DRA goal, and a measure of translocation success. Thirdly, our approach only considered host-specific protozoan and metazoan parasites, at the exclusion of bacteria, fungi, and viruses. As our knowledge of the intricate relationship between the host and its associated microorganisms expands, we will need to be flexible in our application of tools that prioritise the survival of one species over another. Finally, the process for parasite conservation prioritisation we used, whilst based on Carlson et al. (2020; Fig. 1, step 3), was highly simplified, condensing the conservation value of the parasites to a binary question of whether they are host-specific or not. This approach inadequately captures the nuance of parasite conservation decision making (Lymbery and Smit, 2023). We also acknowledge that our decision to conserve host-specific ecto- and endoparasites was relatively straightforward, as the organisms we considered do not cause significant pathology to the host. This decision-making process becomes more challenging when there is conflict between host conservation and parasite conservation objectives. However, considering conservation actions from the lens of both the host and the parasite allows informed decisions to be made.

A limitation of the platypus DRA was that, like the majority of DRAs that have been produced to date, it was generated for a specific project. We believe there are significant benefits to producing national-level, rather than project-centric, DRAs – an approach exemplified by the National Koala Disease Risk Analysis (Vitali et al., 2022). While national-level DRAs are more intensive in terms of time, and involve a larger number of stakeholders, overall, we consider the benefits to outweigh these limitations. Benefits of the national-level approach include encouraging collaboration and coordination of health management and research at a species-level scale, and avoiding the duplication of work each time a DRA is required. Furthermore, this approach consolidates information at a national level, thus producing a more complete understanding of species health and readily identifying knowledge gaps that can subsequently be addressed.

## 5 Conclusion

In conclusion, we found that the streamlined DRA process was readily applied to a platypus translocation, and demonstrated that consideration of parasite conservation can easily be incorporated into the DRA process. Furthermore, we shared learnings from our experience, including challenges related to knowledge gaps and managing the risk posed by geographical barriers. Overall, we believe progressing the incorporation of parasite conservation into conservation translocation planning, including in DRAs, should be considered a priority for translocation practitioners. Future DRA frameworks should embed parasite conservation from the outset, thereby actively promoting ecosystem richness, diversity, and health, and facilitating translocation practitioners to do the same.

## CRediT authorship contribution statement

**Jessica Whinfield:** Conceptualization, Data curation, Formal analysis, Investigation, Methodology, Project administration, Writing – original draft. **Kristin Warren:** Conceptualization, Supervision, Writing – review & editing. **Larry Vogelnest:** Conceptualization, Supervision, Writing – review & editing. **Rebecca Vaughan-Higgins:** Conceptualization, Methodology, Supervision, Writing – review & editing.

## Declaration of competing interest

Declarations of interest: none.
